# Effects of Parecoxib Sodium Application Combined with Enhanced Recovery After Surgery Nursing on Inflammatory Factors and Knee Joint Function in Elderly Patients After Total Knee Arthroplasty

**DOI:** 10.3389/fsurg.2022.902351

**Published:** 2022-06-08

**Authors:** Liqiong Deng, Liping Tan

**Affiliations:** ^1^ Department of Joint Surgery, Chenzhou First People’s Hospital, Chenzhou, China; ^2^ Department of Nursing, Chenzhou First People’s Hospital, Chenzhou, China

**Keywords:** parecoxib sodium, enhanced recovery after surgery, inflammation, knee joint function, total knee arthroplasty

## Abstract

**Objectives:**

To study the effect of parecoxib sodium (PS) application, combined with enhanced recovery after surgery (ERAS) nursing, on inflammation and knee joint function in elderly patients after total knee arthroplasty (TKA).

**Methods:**

In this prospective cross-sectional study, we recruited 120 elderly patients treated with TKA who were randomly divided into two groups, the combine group and the control group, with 60 patients in each group. Patients in the control group received ERAS nursing and normal saline, and the patients in the combine group received ERAS nursing and PS. At different times after surgery, we compared the hemoglobin (Hb), complete white blood cell count (WBC), erythrocyte sedimentation rate (ESR), and serum IL-1β, TNF-α, and IL-6, and recovery time for different ranges of joint motion and the knee joint function HSS (hospital for special surgery scale) score between the two groups.

**Results:**

On the third and seventh postoperative days, the levels of Hb in the patients of the combine group were significantly lower than those in the control group (*p* < 0.05), while the levels of WBC, ESR, serum IL-1β, TNF-α, and IL-6 in the patients of the combine group were all significantly lower than those in the control group (*p* < 0.05). Compared with the patients in the control group, the recovery time for 30, 60, 90, and 120 angles of joint motion in patients of the combine group was significantly decreased (*p* < 0.05), and the HSS score of patients in the combine group was significantly higher than that in the control group on the first, third, and sixth postoperative months (*p* < 0.05).

**Conclusion:**

Elderly TKA patients who received PS application, combined with ERAS nursing, had lower inflammation in peripheral blood 2 weeks after operation and faster postoperative recovery of knee joint function.

## Introduction

Total knee arthroplasty (TKA) is the main program for the treatment of severe knee joint diseases, and it can relieve knee joint pain and rebuild knee joint function through surgery (1, 2). However, 15%–20% of patients after TKA are dissatisfied with analgesia because they cannot perform functional exercise due to pain, there is delayed discharge, and they are prone to complications such as deep vein thrombosis, pulmonary embolism, infection, and postoperative ankylosis. Therefore, scientific nursing and analgesic protocols are valuable for patients receiving TKA (3, 4). In addition, severe pain after TKA makes it difficult for many patients to participate in early postoperative rehabilitation and exercise, which may lead to unsatisfactory recovery of knee joint function and greatly reduce the quality of life of the patients (5). Perioperative pain directly affects postoperative recovery and surgical outcome. Therefore, a scientifically rational model of care and analgesic administration will be valuable for TKA patients.

Enhanced recovery after surgery (ERAS) nursing is a series of perioperative optimization measures based on evidence-based medicine to reduce the body’s stress response, promote postoperative recovery of patients, shorten hospital stay, reduce postoperative complications, and reduce readmission risk and the risk of death (6, 7). However, the degree of pain severely restricts the implementation of ERAS, affecting important evaluation indicators such as joint range of motion, time to start functional exercise, and average hospital stay (8). Therefore, TKA perioperative analgesia is an important option that needs to be popularized under the ERAS concept. For patients undergoing surgical procedures, there are many analgesic protocols that are currently available, including preemptive analgesia (9–11). Preemptive analgesia is the administration of a certain dose of analgesics before the transmission of pain stimuli to block nociceptive transmission and achieve the purpose of reducing postoperative pain. Preemptive analgesia protocols is the administration of a certain dose of analgesics before the transmission of pain stimuli to block nociceptive transmission and achieve the purpose of reducing postoperative pain (12, 13). PS is a specific COX-2 inhibitor that can inhibit peripheral COX-2 expression and reduce the synthesis of peripheral prostaglandins (PG), thereby exerting analgesic and anti-inflammatory effects (14, 15). At the same time, PS can also inhibit the expression of central COX-2, reduce the synthesis of central PG, significantly reduce the level of PGE2 in the cerebrospinal fluid, reduce central sensitization, and exert dual analgesic effects (14, 15).

In this prospective cross-sectional study, we aim to ascertain the effect of PS application, combined with ERAS, on inflammation and knee joint function in elderly patients after TKA.

## Materials and Methods

### Patients and Ethics Statement

The present study is approved and supervised by our Hospital Ethics Committee, and it conforms to the principles of the Declaration of Helsinki. Moreover, all volunteers who participated in this study were briefed about its content, following which they signed an informed consent form.

In this prospective cross-sectional study, we enrolled 120 elderly patients treated with TKA from January 2020 to September 2021. The inclusion criteria were as follows: (1) age >60 years; (2) treated with TKA; (3) ASA stages I–III; (4) weight range from 45 to 90 kg; and (5) complete clinical data and voluntary participation in this study. The exclusion criteria were as follows: (1) coagulation dysfunction; (2) liver, kidney, or other tissue and organ dysfunction; (3) patients with immune system diseases, tumors, and chronic infectious diseases; (4) history of central and peripheral nervous system disease; (5) history of drug abuse, alcohol abuse, opioid abuse, non-steroidal anti-inflammatory drug allergy; (6) surgery within 1 year; and (7) intellectual disability or mental illness.

### Parecoxib Sodium Administration

In this study, all patients who had undergone TKA treatment received ERAS nursing. However, patients in the combine group received PS administration. The protocol of PS administration was as follows: After completing the anesthesia protocol and before preparing to cut the skin, the patients in the combine group were injected with 40- mg PS intravenously. At 12, 24, 36, and 48 h after TKA treatment, these patients were again injected with 40- mg PS intravenously. Patients in the control group were intravenously injected with an equal volume of normal saline at the same time. The protocol of ERAS nursing was as follows (2 weeks): (1) Dorsiflexion training: dorsiflexion exercise 6 h after operation, dorsiflexion for 5 s, and then plantar flexion for 5 s and 100 times/h; (2) Walking on the ground: walking out of bed on the second day after operation, 1 time/2 h and 10 min/time; (3) Knee joint flexion and extension exercise: On the third day after operation, the affected limb knee joint extension exercise and flexion exercise were performed on the bed, both 1 time/2 h and 10 min/time second rate.

### Hematology Indicators

Before surgery and 1, 3, 7, and 14 days after surgery, 10 ml of peripheral blood was collected from each patient to detect the levels of hemoglobin (Hb), complete white blood cell count (WBC), erythrocyte sedimentation rate (ESR), and serum IL-1β, TNF-α, and IL-6.

### Knee Joint Function

Before surgery and first, third, and sixth months after surgery, we used the hospital for special surgery scale (HSS) to evaluate the knee joint function. The HSS score includes the following 7 sub-items: pain, function, range of motion, muscle strength, flexion deformity, stability, and deduction items. Higher HSS scores indicate better knee function. At the same time, we recorded the recovery time for 30, 60, 90, and 120 angles of joint motion in patients with TKA treatment.

### Statistical Analysis

SPSS 20.0 (NIH, USA) was used to analyze the data in the present study. Measurement data that conform to a normal distribution pattern are presented as (mean ± standard deviation), and the difference in the measurement data between the two groups is compared using an independent-samples *t*-test. Categorical data are presented as numbers and percentages and analyzed by Chi-squared analysis or the Fisher exact-probability test. A score of *p* < 0.05 was considered statistically significant.

## Results

### Baseline Data

We compared the baseline data including gender, age, BMI, ASA grade, surgical, and surgical time between the combine group and the control group and found no significant difference (Table 1).

**Table 1:** Comparison of baseline data of two groups of patients (*n*, $\bar{x}\pm s$).

Index	Combine group (*n* = 60)	Control group (*n* = 60)	*t*/*χ*^2^	*p*
Gender (*n*)				
Male	28	30	0.075	0.726
Female	32	30		
Age (years)	64.3 ± 7.9	65.2 ± 9.4	0.537	0.715
BMI (kg/m^2^)	24.6 ± 1.3	24.5 ± 1.2	1.021	0.191
ASA Grade				
I + II	38	34	0.278	0.598
III	22	26		
Surgical site				
Right	26	29	1.004	0.316
Left	24	21		
Surgical time (min)	80.2 ± 10.3	79.8 ± 9.5	0.631	0.602

### Peripheral Blood Inflammation–Related Indicators

**Figure 1:**
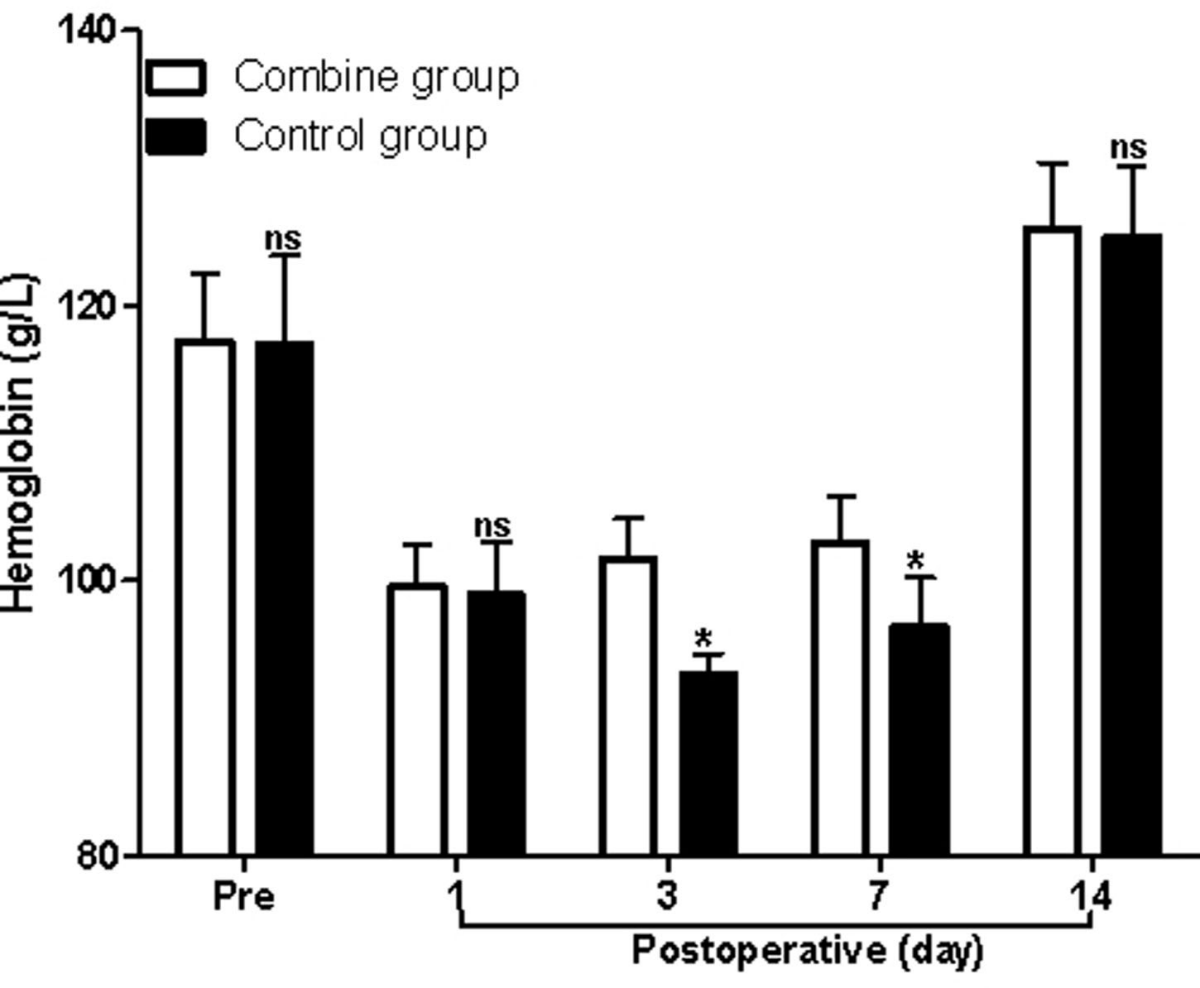
Comparison of hemoglobin at different times in patients of two groups. Note: Compared with the combine group, ^ns^*p* > 0.05 and *p < 0.05.

**Figure 2:**
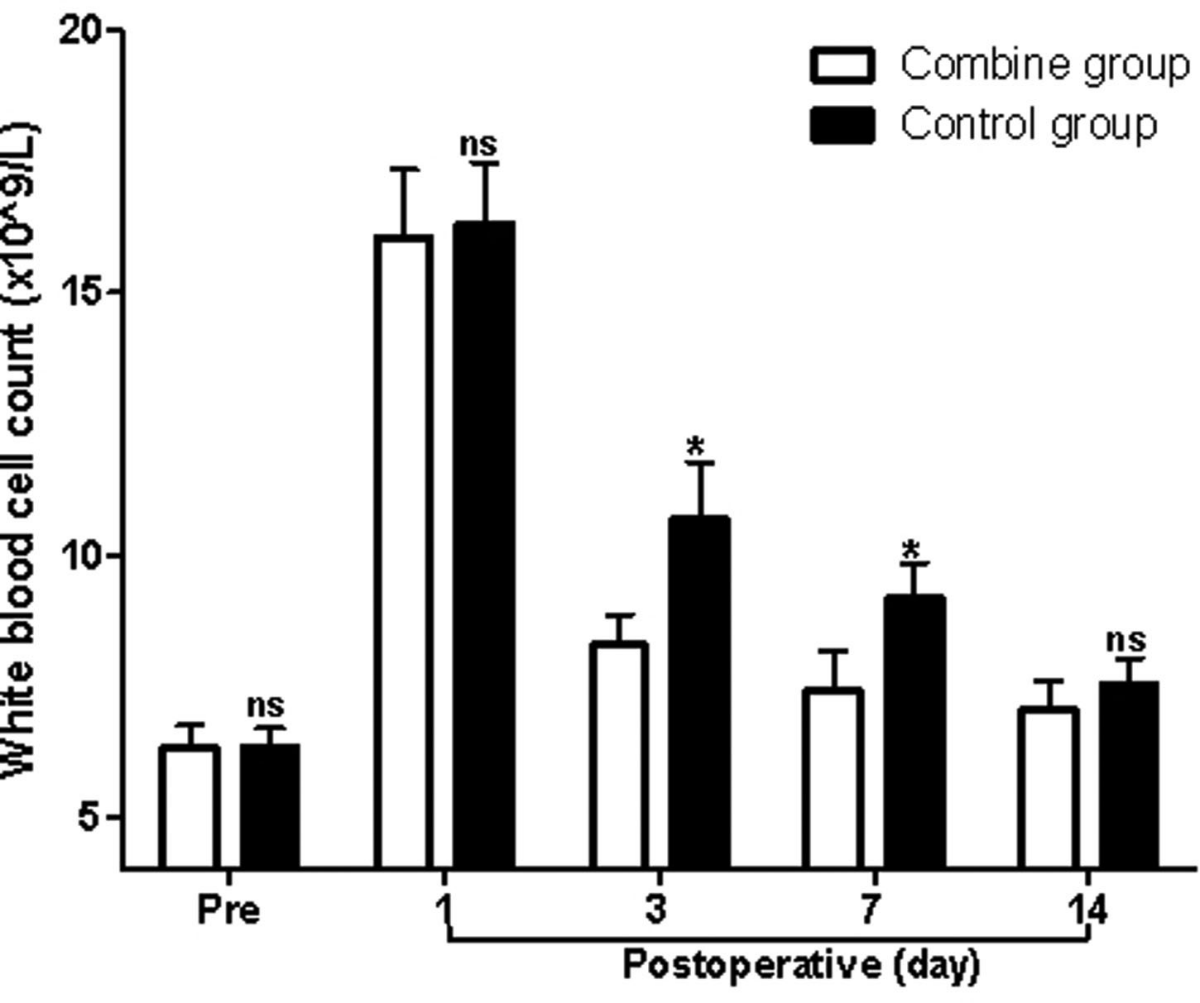
Comparison of white blood cell count at different times in patients of two groups. Note: Compared with the combine group, ^ns^*p* > 0.05 and *p < 0.05.

**Figure 3:**
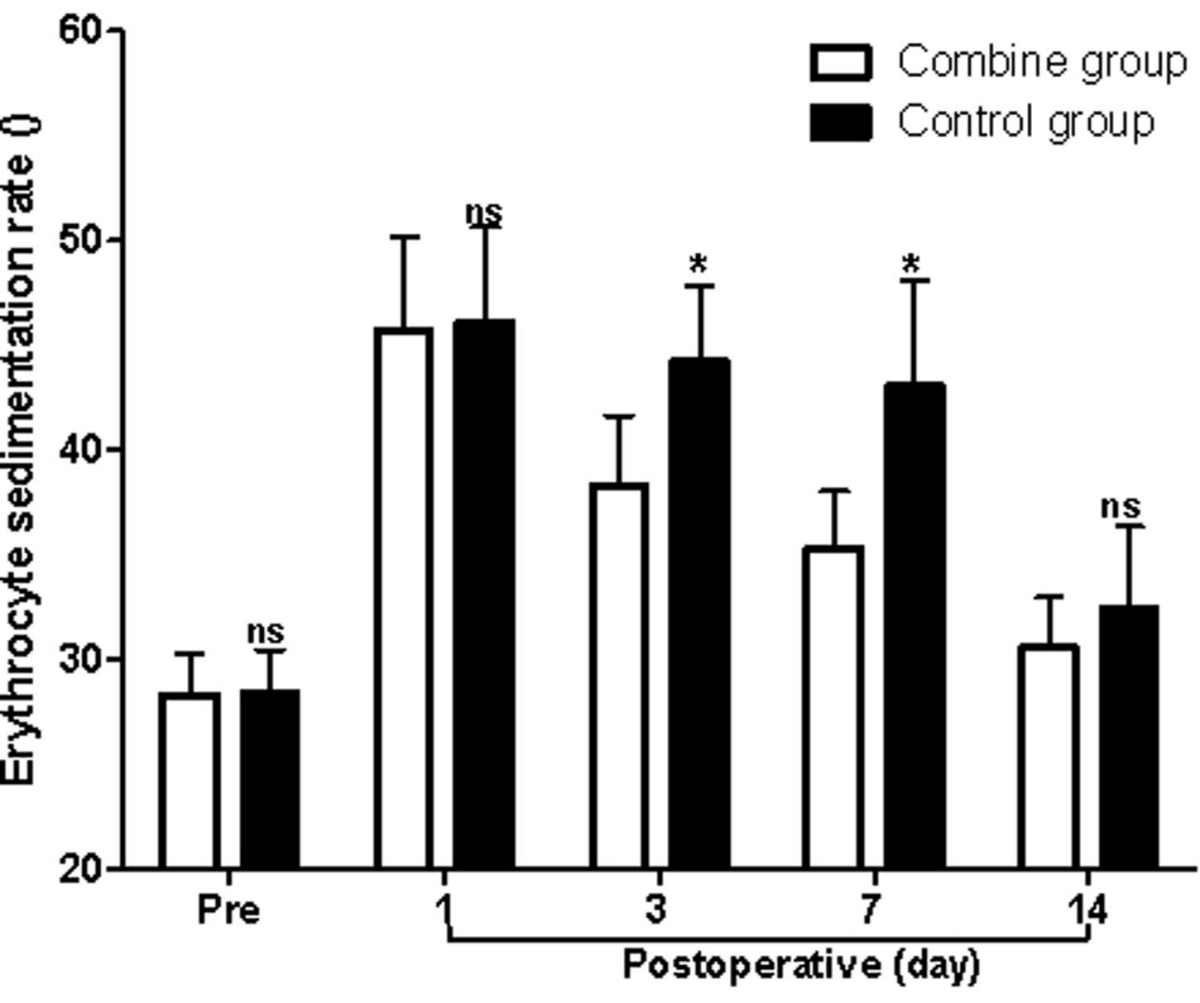
Comparison of the erythrocyte sedimentation rate at different times in patients of two groups. Note: Compared with the combine group, ^ns^*p* > 0.05 and *p < 0.05.

At first, before surgery, the levels of Hb, WBC, and ESR in the patients of the combine group showed no significant difference compared with those of the control group (*p* > 0.05). On the third and seventh postoperative days, the levels of Hb in the patients of the combine group were significantly higher than those of the control group (*p* < 0.05; Figure 1), while the levels of WBC and ESR in the patients of the combine group were significantly lower than those of the control group (*p* < 0.05; Figures 2 and 3). On the 1st and 14th postoperative days, the levels of Hb, WBC, and ESR in the patients of the combine group showed no significant difference compared with those of the control group (*p* > 0.05).

### Serum Inflammatory Cytokines

**Figure 4:**
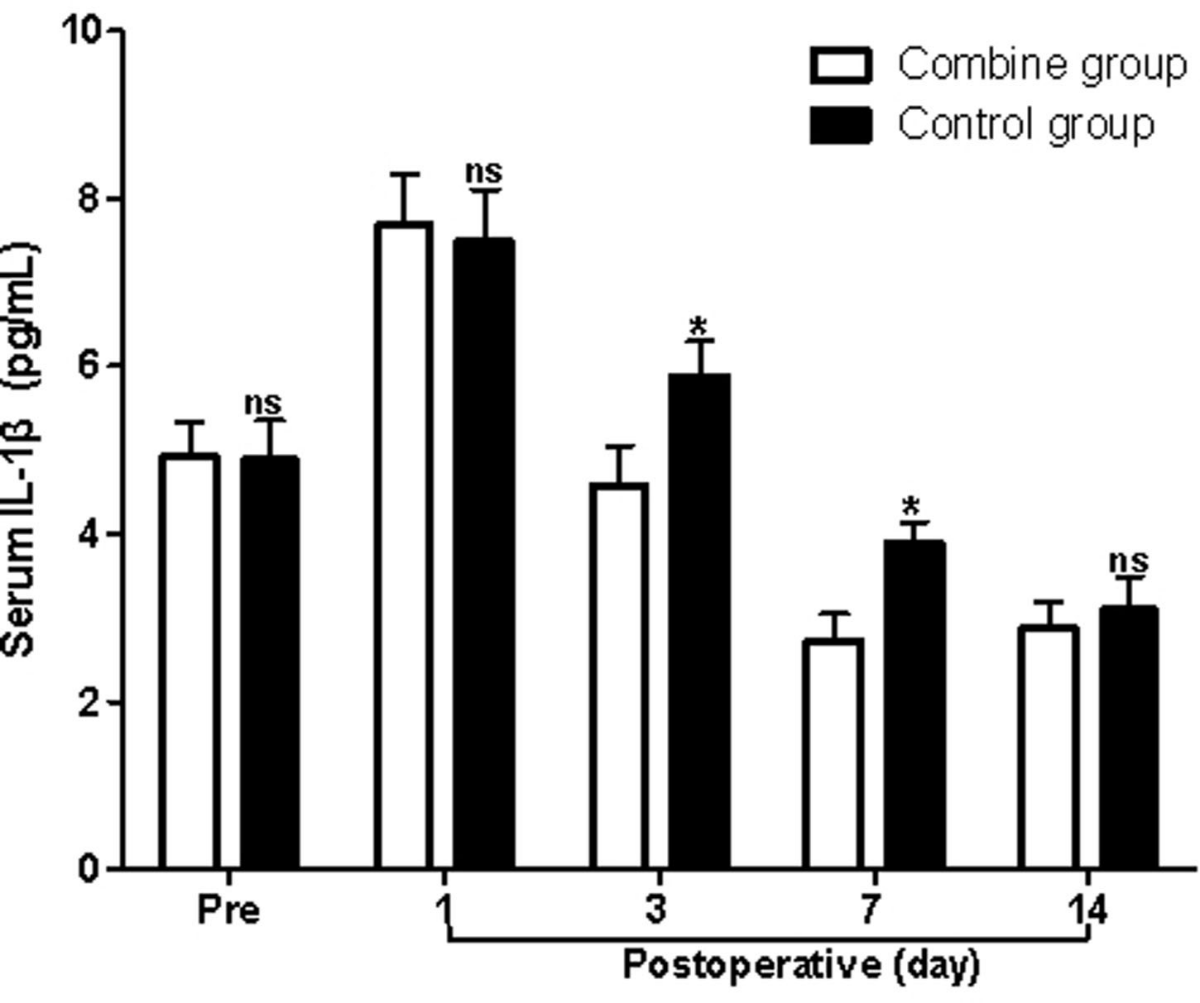
Comparison of serum IL-1β levels at different times in patients of two groups. Note: Compared with the combine group, ^ns^*p* > 0.05 and *p < 0.05.

**Figure 5:**
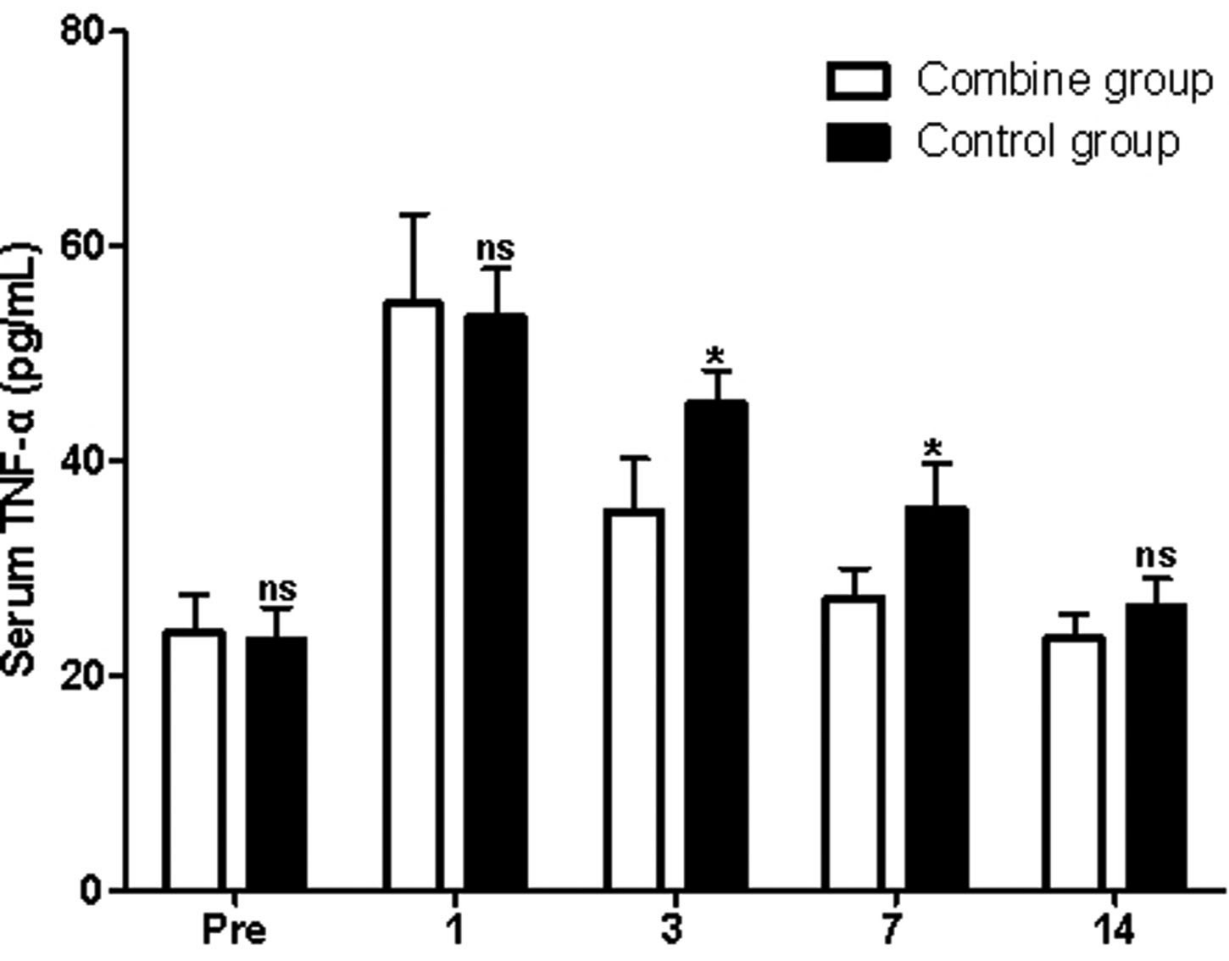
Comparison of serum TNF-α levels at different times in patients of two groups. Note: Compared with the combine group, ^ns^*p* > 0.05 and *p < 0.05.

**Figure 6:**
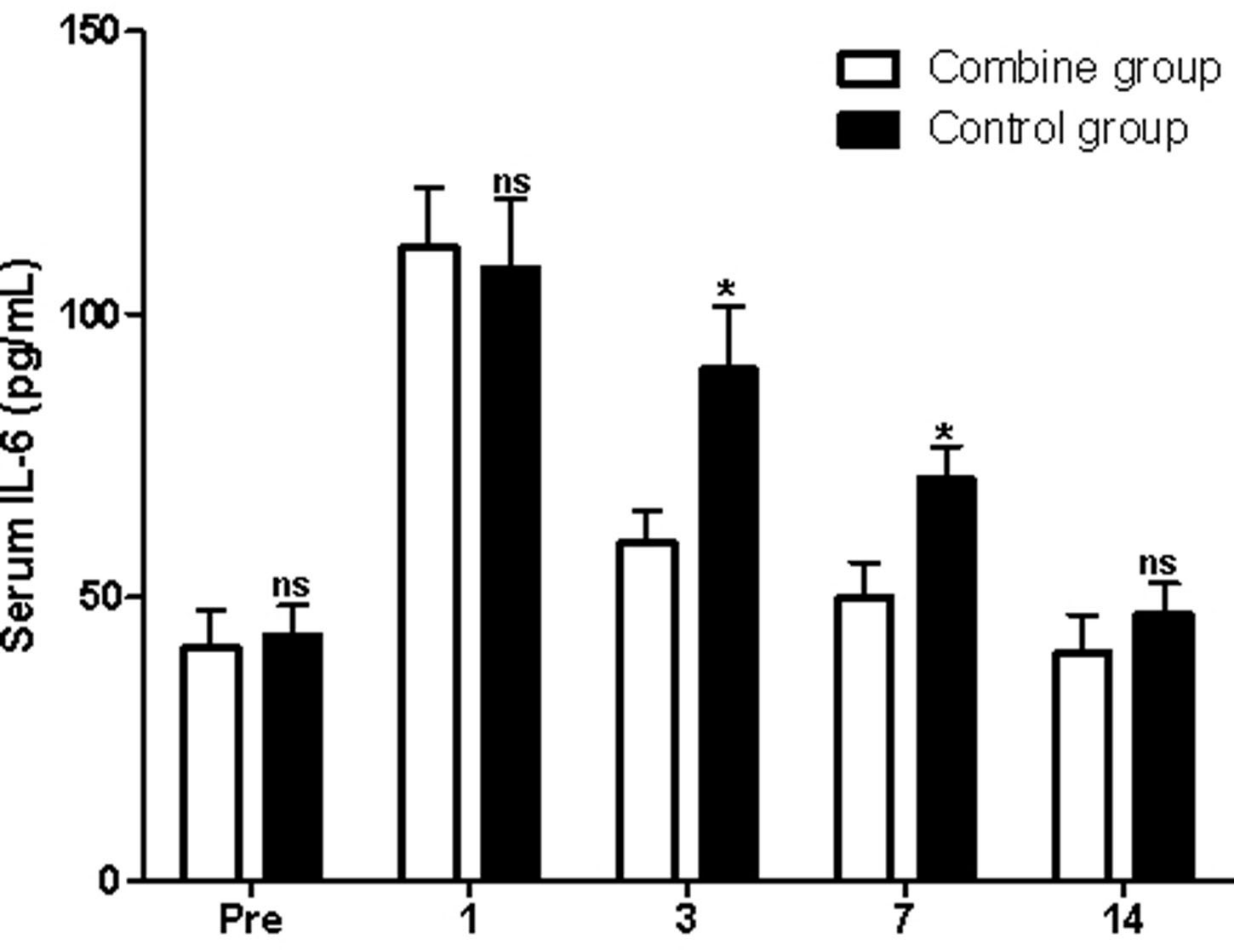
Comparison of serum IL-6 levels at different times in patients of two groups. Note: Compared with the combine group, ^ns^*p* > 0.05 and *p < 0.05.

At first, before surgery, the serum levels of IL-1β, TNF-α, and IL-6 in the patients of the combine group showed no significant difference compared with those of the control group (*p* > 0.05). On the third and seventh postoperative days, the serum levels of IL-1β, TNF-α, and IL-6 in the patients of the combine group were all significantly lower than those of the control group (*p* > 0.05; Figures 4–6), while there was no significant difference on the 1st and 14th postoperative days (*p* > 0.05).

### Recovery Time for Different Ranges of Joint Motion

Compared with patients in the control group, the recovery time for 30, 60, 90, and 120 angles of joint motion in patients of the combine group was significantly decreased (*p* < 0.05; Table 2).

**Table 2:** Comparison of recovery time for different ranges of joint motion in patients of two groups (*n*, $\bar{x}\pm s$).

Group	*n*	Range of motion (°)			
		30 (days)	60 (days)	90 (days)	120 (days)
Combine group	60	1.0 ± 0.3	6.0 ± 2.1	20.5 ± 3.8	28.2 ± 4.8
Control group	60	6.5 ± 1.8	13.7 ± 4.3	27.5 ± 4.8	40.1 ± 6.4
*t*		6.592	7.326	9.526	13.824
*p*		<0.001	<0.001	<0.001	<0.001

### Knee Joint Function HSS Score

Before surgery, the HSS score of patients in the combine group showed no significant difference from that of patients in the control group (*p* > 0.05). On the first, third, and sixth postoperative months, the HSS score of patients in the combine group was significantly higher than that in the control group (*p* < 0.05; Table 3).

**Table 3:** Comparison of the HSS score at different times in patients of two groups (*n*, $\bar{x}\pm s$, scores).

Group	*n*	Preoperative	Postoperative (month)		
			1	3	6
Combine group	60	45.6 ± 9.8	65.8 ± 9.3	75.6 ± 5.8	86.5 ± 6.2
Control group	60	45.9 ± 9.6	56.2 ± 7.5	68.2 ± 5.9	77.5 ± 7.1
*t*		0.786	5.326	3.291	2.913
*p*		0.382	<0.001	0.026	0.039

## Discussion

Degenerative knee arthritis is a disease with a high incidence among the elderly people, and the clinical features are mainly redness, swelling, pain, and snapping of the knee joint (16, 17). If patients cannot receive timely treatment, it will lead to severe joint deformity and even disability. The pathogenesis of degenerative joint disease is related to advanced age, obesity, trauma, genetic, and metabolic factors. At present, in the early stage of the disease, conservative treatment is mainly used to delay the development of the disease, while in the middle and late stages of the disease, when the knee joint function is lost due to joint pain, deformity, etc., only a part of the joint function can be restored through knee joint replacement and other methods (18). Currently, TKA is a common method for the treatment of severe knee osteoarthritis (18). However, TKA is more traumatic to patients with knee osteoarthritis, and severe inflammatory reaction occurs after surgery and releases pain-causing substances, causing postoperative pain and affecting the recovery of postoperative joint function (5). Perioperative analgesia can help reduce the inflammatory response caused by TKA, reduce tissue exudation and edema, reduce perioperative hidden and overt bleeding, promote wound healing, and accelerate the functional recovery of the affected knee joint (9–11).

Severe inflammatory and release of pain-causing substances will occur after TKA, which are the main inducers of postoperative pain in the body (19, 20). PS is a new type of non-steroidal anti-inflammatory drug with analgesic, antipyretic, anti-inflammatory, and anti-rheumatic effects. PS is suitable for pain management of various acute and chronic inflammatory arthritis and is widely used in perioperative multimodal preemptive analgesia (14, 15). In addition, parecoxib is a prodrug of valdecoxib. On the one hand, it can reduce the synthesis of PG by inhibiting the activity of COX-2 in the peripheral and central regions, thereby reducing peripheral and central sensitization (14, 15). On the other hand, it exerts the analgesic effect by reducing the postoperative inflammatory response and shortening the duration of this response (21, 22).

In the present study, patients in the combine group received ERAS nursing and PS, while patients in the control group received ERAS nursing and normal saline. We found that the levels of Hb in the patients of the combine group were significantly higher than those of the control group, while the levels of WBC, ESR, serum IL-1β, TNF-α, and IL-6 in the patients of the combine group were all significantly lower than those of the control group on the third and seventh postoperative days, which suggested that patients in the combine group had lower postoperative inflammation in peripheral blood. Tissue damage caused by TKA can activate the complement system and immune cells, causing the body to release a variety of inflammatory factors such as IL-1β, IL-6, and TNF-α (23–26). IL-1β and IL-6 are the main pro-inflammatory factors of acute inflammatory response and play an important role in regulating body damage, infection, etc. The levels of IL-1β and IL-6 increase rapidly under stress, which is closely related to the degree of tissue damage (23–26).

The recovery time of postoperative knee joint function in patients receiving TKA is the gold standard for evaluating the efficacy of postoperative analgesia and nursing modes. In this study, we found that the recovery time for 30, 60, 90, and 120 angles of joint motion in patients of the combine group was significantly lower than that of the control group, which suggested that patients in the combine group showed a faster postoperative recovery of knee joint function. Moreover, we found that the HSS score of patients in the combine group was significantly higher than that in the control group on the first, third, and sixth postoperative months, which also that suggested patients in the combine group showed a faster postoperative recovery of knee joint function. Lesser postoperative inflammation in peripheral blood leads to lesser postoperative pain, that is, a better analgesic effect, and a better analgesic effect helps to reduce the inflammatory reaction caused by TKA and also reduce tissue exudation and edema. It can reduce the amount of hidden and dominant bleeding in the perioperative period, promote wound healing, and accelerate the functional recovery of the affected knee joint (27–29).

## Conclusion

Elderly TKA patients who received PS application, combined with ERAS, had lower inflammation in peripheral blood 2 weeks after operation and showed faster postoperative recovery of knee joint function.

## Data Availability

The original contributions presented in the study are included in the article/Supplementary Material; further inquiries can be directed to the corresponding author/s.

## References

[1] Alesi D Meena A Fratini S Rinaldi VG Cammisa E Lullini G Total knee arthroplasty in valgus knee deformity: is it still a challenge in 2021? Musculoskelet Surg. (2022) 106(1):1–8. 10.1007/s12306-021-00695-x 33587251 PMC8881420

[2] Alrawashdeh W Eschweiler J Migliorini F El Mansy Y Tingart M Rath B. Effectiveness of total knee arthroplasty rehabilitation programmes: a systematic review and meta-analysis. J Rehabil Med. (2021) 53(6):jrm00200. 10.2340/16501977-2827 33846757 PMC8814866

[3] Rutherford RW Jennings JM Dennis DA. Enhancing recovery after total knee arthroplasty. Orthop Clin North Am. (2017) 48(4):391–400. 10.1016/j.ocl.2017.05.002 28870300

[4] Li JW Ma YS Xiao LK. Postoperative pain management in total knee arthroplasty. Orthop Surg. (2019) 11(5):755–61. 10.1111/os.12535 31663286 PMC6819170

[5] Gaffney CJ Pelt CE Gililland JM Peters CL. Perioperative pain management in hip and knee arthroplasty. Orthop Clin North Am. (2017) 48(4):407–19. 10.1016/j.ocl.2017.05.001 28870302

[6] Smith TW Jr Wang X Singer MA Godellas CV Vaince FT. Enhanced recovery after surgery: a clinical review of implementation across multiple surgical subspecialties. Am J Surg. (2020) 219(3):530–4. 10.1016/j.amjsurg.2019.11.009 31761300

[7] Nelson G Bakkum-Gamez J Kalogera E Glaser G Altman A Meyer LA Guidelines for perioperative care in gynecologic/oncology: Enhanced Recovery After Surgery (ERAS) Society recommendations-2019 update. Int J Gynecol Cancer. (2019) 29(4):651–68. 10.1136/ijgc-2019-000356 30877144

[8] Kaye AD Chernobylsky DJ Thakur P Siddaiah H Kaye RJ Eng LK Dexmedetomidine in Enhanced Recovery After Surgery (ERAS) protocols for postoperative pain. Curr Pain Headache Rep. (2020) 24(5):21. 10.1007/s11916-020-00853-z 32240402 PMC7223065

[9] Beverly A Kaye AD Ljungqvist O Urman RD. Essential elements of multimodal analgesia in Enhanced Recovery After Surgery (ERAS) guidelines. Anesthesiol Clin. (2017) 35(2):e115–43. 10.1016/j.anclin.2017.01.018 28526156

[10] Chanques G Constantin JM Devlin JW Ely EW Fraser GL Gélinas C Analgesia and sedation in patients with ARDS. Intensive Care Med. (2020) 46(12):2342–56. 10.1007/s00134-020-06307-9 33170331 PMC7653978

[11] Byrne K Smith C. Preemptive analgesia: an unobtainable goal? J Cardiothorac Vasc Anesth. (2019) 33(2):460–1. 10.1053/j.jvca.2018.08.008 30217585

[12] Azevedo I Ugalde Figueroa P. Commentary: Can preemptive analgesia decrease opioid use after foregut laparoscopic surgery? J Thorac Cardiovasc Surg. (2020) 159(2):747–8. 10.1016/j.jtcvs.2019.06.072 31378405

[13] Zhang LK Li Q Quan RF Liu JS. Is preemptive analgesia a good choice for postoperative pain relief in lumbar spine surgeries?: A meta-analysis of randomized controlled trials. Medicine (Baltimore). (2021) 100(13):e25319. 10.1097/MD.0000000000025319 33787624 PMC8021355

[14] Huang Z Ma X Jia X Wang R Liu L Zhang M Prevention of severe acute pancreatitis with cyclooxygenase-2 inhibitors: a randomized controlled clinical trial. Am J Gastroenterol. (2020) 115(3):473–80. 10.14309/ajg.0000000000000529 32142484 PMC7060052

[15] Zhuang Q Tao L Lin J Jin J Qian W Bian Y Postoperative intravenous parecoxib sodium followed by oral celecoxib post total knee arthroplasty in osteoarthritis patients (PIPFORCE): a multicentre, double-blind, randomised, placebo-controlled trial. BMJ Open. (2020) 10(1):e030501. 10.1136/bmjopen-2019-030501 31924632 PMC6955469

[16] Mahmoudian A Lohmander LS Mobasheri A Englund M Luyten FP. Early-stage symptomatic osteoarthritis of the knee - time for action. Nat Rev Rheumatol. (2021) 17(10):621–32. 10.1038/s41584-021-00673-4 34465902

[17] Jang S Lee K Ju JH. Recent updates of diagnosis, pathophysiology, and treatment on osteoarthritis of the knee. Int J Mol Sci. (2021) 22(5):2619. 10.3390/ijms22052619 33807695 PMC7961389

[18] Quinn RH Murray JN Pezold R Sevarino KS. Surgical management of osteoarthritis of the knee. J Am Acad Orthop Surg. (2018) 26(9):e191–3. 10.5435/JAAOS-D-17-00424 29688919

[19] Dyskova T Kriegova E Slobodova Z Zehnalova S Kudelka M Schneiderova P Inflammation time-axis in aseptic loosening of total knee arthroplasty: a preliminary study. PLoS ONE. (2019) 14(8):e0221056. 10.1371/journal.pone.0221056 31469844 PMC6716666

[20] Jang JS Choi WK. Factors affecting the duration of antibiotic use due to surgical site inflammation after complication-free classical total knee arthroplasty. Medicine. (2022) 101(4):e28605. 10.1097/MD.0000000000028605 35089196 PMC8797555

[21] Chong SJ Wong YC Wu J Tan MH Lu J Moochhala SM Parecoxib reduces systemic inflammation and acute lung injury in burned animals with delayed fluid resuscitation. Int J Inflam. (2014) 2014:972645. 10.1155/2014/972645 24579056 PMC3918702

[22] Zhu Y Wang S Wu H Wu Y. Effect of perioperative parecoxib on postoperative pain and local inflammation factors PGE2 and IL-6 for total knee arthroplasty: a randomized, double-blind, placebo-controlled study. Eur J Orthop Surg Traumatol. (2014) 24(3):395–401. 10.1007/s00590-013-1203-4 23483320

[23] Eroğlu M Kokulu S Koca HB Demirbogan ME Baki ED Özcan Ö The effects of general and spinal anesthesia on systemic inflammatory response in patients undergoing total knee arthroplasty. Eklem Hastalik Cerrahisi. (2016) 27(3):153–9. 10.5606/ehc.2016.31 27902170

[24] Islam N Whitehouse M Mehendale S Hall M Tierney J O'Connell E Post-traumatic immunosuppression is reversed by anti-coagulated salvaged blood transfusion: deductions from studying immune status after knee arthroplasty. Clin Exp Immunol. (2014) 177(2):509–20. 10.1111/cei.12351 24749651 PMC4226602

[25] Martin F Martinez V Mazoit JX Bouhassira D Cherif K Gentili ME Antiinflammatory effect of peripheral nerve blocks after knee surgery: clinical and biologic evaluation. Anesthesiology. (2008) 109(3):484–90. 10.1097/ALN.0b013e318182c2a1 18719447 PMC2758602

[26] Chloropoulou P Iatrou C Vogiatzaki T Kotsianidis I Trypsianis G Tsigalou C Epidural anesthesia followed by epidural analgesia produces less inflammatory response than spinal anesthesia followed by intravenous morphine analgesia in patients with total knee arthroplasty. Med Sci Monit. (2013) 19:73–80. 10.12659/MSM.883749 23353589 PMC3628992

[27] Wu L Si H Li M Zeng Y Wu Y Liu Y The optimal dosage, route and timing of glucocorticoids administration for improving knee function, pain and inflammation in primary total knee arthroplasty: a systematic review and network meta-analysis of 34 randomized trials. Int J Surg. (2020) 82:182–91. 10.1016/j.ijsu.2020.07.065 32877755

[28] Prince N Penatzer JA Dietz MJ Boyd JW. Localized cytokine responses to total knee arthroplasty and total knee revision complications. J Transl Med. (2020) 18(1):330. 10.1186/s12967-020-02510-w 32867801 PMC7461261

[29] Lin S Wu B Li Y. A commentary on “The optimal dosage, route and timing of glucocorticoids administration for improving knee function, pain and inflammation in primary total knee arthroplasty: a systematic review and network meta-analysis of 34 randomized trials”. Int J Surg. (2021) 87:105888. 10.1016/j.ijsu.2021.01.014 33545370

